# Framing the Default: Influence of Choosing Versus Rejecting Frame on Default Effects

**DOI:** 10.1027/1618-3169/a000617

**Published:** 2024-10-09

**Authors:** Subramanya P. Chandrashekar, Adrien A. Fillon

**Affiliations:** ^1^ Department of Psychology, Norwegian University of Science and Technology (NTNU), Trondheim, Norway; ^2^ ERA Chair in Science and Innovation Policy & Studies (SInnoPSis), University of Cyprus, Nicosia, Cyprus

**Keywords:** default effect, framing effects, status quo, nudge, choice architecture

## Abstract

**Abstract:** A key finding within nudging research is the *default effect*, where individuals are inclined to stay with a default option when faced with a decision, rather than exploring alternatives (e.g., a preselected job opportunity among two alternatives). Similarly, the study of framing effects delves into how the presentation and context of decisions influence choices (e.g., choosing vs. rejecting a job opportunity among two alternatives). Specifically, previous literature examining *choosing versus rejecting* decision frames in various situations has found that these frames do not invariably complement each other; therefore, individuals’ preferences vary based on the task frame. Yet, simultaneous testing of multiple nudges remains relatively unexplored in the literature. In the current study involving 1,072 participants, we examined how framing and default effects can influence decision-making in hypothetical scenarios. The decision scenarios involved two different domains—work and health. We found that framing had a strong effect on decision-making in both work and health domains, whereas default setting contributed only to a limited extent in the work domain and no effect was found in the health domain, mirroring related recent research findings. We argue for a more careful design of nudge interventions when multiple overlapping nudges are used and for a contextual approach to applying behavioral science to citizens.


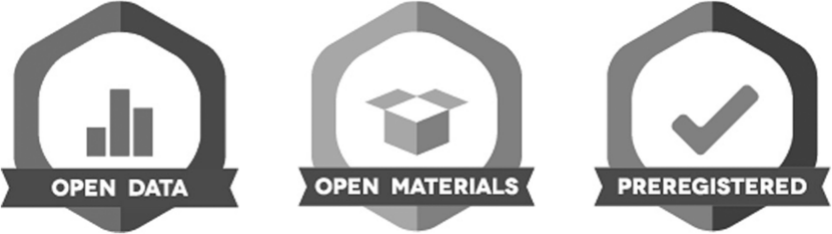


The default effect refers to the tendency of people to stick with the pre-selected option when given a choice. For example, if an online form has a box already checked to receive newsletters, most people would not uncheck it, thereby opting in by default. Similarly, a default effect in organ donation refers to the phenomenon where people are more likely to become organ donors when the default option is to be an organ donor (referred to as an opt-out system) rather than when they have to actively choose to become a donor (opt-in system). Making organ donation a default option has led to the promise to save thousands of lives (Johnson & Goldstein, 2003).

Like the example noted above, default effects are a category of *behavioral nudges* – subtle interventions designed to influence people’s decisions without restricting their choices. Recent broader critiques of behavioral nudges include that these suffer from publication bias (Maier et al., 2022; Szaszi et al., 2022). Jachimowicz et al.’s (2019) meta-analysis notes substantial variations in default effect sizes. Additionally, DellaVigna and Linos (2022) found larger effect sizes for nudge interventions reported in published literature than those reported by Nudge Units^1^ in the United States. This suggests that selective reporting can even inflate meta-analytic effect sizes (Kvarven et al., 2020). Moreover, in some cases, nudge effects did not replicate with large samples (Bohner & Schlüter, 2014; Kettle et al., 2017; Kristal et al., 2020).

So far, a few published works have documented inconsistent findings in the literature. Narula et al. (2014) investigated the influence of default effects in the context of preventive health appointments and discovered results contrary to the predictions of the default effect. Specifically, participants in an opt-in condition exhibited a higher show-up rate for health appointments compared to participants in an opt-out condition. A similar pattern emerged in the study by Reiter et al. (2012) when examining consent rates for Human Papillomavirus (HPV) vaccinations. Additionally, several studies have reported null findings (Abhyankar et al., 2014; Everett et al., 2015; Jin, 2011; Löfgren et al., 2012). However, most of the published literature continues to support the predictions of the default effect (see Jachimowicz et al., 2019 for a review).

In summary, the majority of published literature support the predictions of the default effects, although few studies document the opposite pattern. Field studies involving a large sample of participants and measuring behavior suggest mixed support for default effect and importantly smaller effect sizes on average than published literature that mainly involves lab studies. There are a few possible ways to explain the inconsistencies in the findings. One such possibility is the predominance of large effects in the published literature suggests publication bias, as it is easier to publish a finding that supports an established set of findings than null findings. The second possibility is variations in the effect sizes suggest a need for studies on contextual influence on default effects. The lab studies have a high internal validity and can control for other contextual influences that are not possible in field studies environment. Therefore, default effects observed in the field are informed by some contextual influences.

The robustness of the default effect amid contextual factors has received limited research attention, except for Johnson et al. (2002), who explored it in an online subscription scenario. Johnson et al. (2002) examined how people arrive at a choice by contextually varying whether participants were asked to choose an alternative or reject an alternative in the online subscription scenario, all while manipulating the default (opt-in vs. opt-out). Such a framing of the choice scenario is referred to as framing effects. Johnson et al. (2002) argued for separate effects of framing and defaults, a recent replication (Chandrashekar et al., 2023) contradicts this, emphasizing framing's significant influence on default effectiveness. This challenges the notion of additive effects, suggesting default effects are sensitive to contexts. These findings highlight framing's crucial role in default effects, urging careful consideration. Exploring framing as a contextual factor not only enhances our understanding of when defaults matter but also elucidates their boundaries. Recognizing contextual nuances allows us to devise interventions and policies that harness default effects positively. Thus, our study aims to deepen insights into framing's impact on default nudges, exploring conditions influencing this interaction and strategies for enhancing default effectiveness.

## The Aims of the Present Study

The present work has two important goals. First, we examine default effects in a decision context that differs from typical decision scenarios studied in the literature. For example, the organ donation scenario involves a dichotomous decision – to consent or not. In the present study, the decision scenarios entail a comparative evaluation of two options, such as product A versus product B. Secondly, and most importantly, we explore the implications of multiple nudge interventions simultaneously, i.e., we consider default effects together with framing effects (choosing vs. rejecting frames). This aspect is crucial for both theoretical advancement and practical application. Theoretically, this approach can further our understanding of how nudges can potentially complement or conflict with one another. Take the example of the default effect; there is supporting evidence in the academic literature that people stick with the default choice. However, a person approaching a decision scenario can view it as a selection versus rejection process. Such variations in the approach are ecologically valid as people can be more prone (trait-like) or situationally prompted to view a decision scenario as choosing versus rejection. Practically, understanding these interactions can also guide the development of a more nuanced framework for behavioral intervention.

In the following sections of the paper, we provide a theoretical overview of default effects and framing effects, which will form the basis for the proposed empirical study.

## The Role of Framing in Default Choices

### The Default Effect

How options are presented affects people’s choices (Kahneman & Tversky, 1984; Tversky & Kahneman, 1981; Tversky et al., 1988). For instance, when a choice is presented with a default option among other possible options, people are more likely to prefer the default (Johnson et al., 2002). Published literature predominantly document support for the default effect across various domains, such as health, retirement saving, organ donation, sustainability, insurance coverage, electricity consumption, and charitable giving (Abadie & Gay, 2006; Benartzi & Thaler, 1999; Cronqvist & Thaler, 2004; Madrian & Shea, 2001; Shealy & Klotz, 2015). A recent meta-analysis of default effects in the published literature reports an aggregated effect size of, *d* = 0.68, 95% CI [0.53, 0.83] (Jachimowicz et al., 2019).

A few explanations for the default effect have been suggested. First, some argue that sticking with the default is best explained by effort (McKenzie et al., 2006). Sticking with the default requires little or no effort, while changing from the default option may require relatively more effort. If people want to minimize the cognitive effort in a decision, they will stick with the default option rather than actively change the default (cf., Johnson & Goldstein, 2003). Second, decision-makers assume that defaulted alternatives are endorsements or recommendations by the question-poser (Brown & Krishna, 2004; McKenzie et al., 2006). A third explanation for the default effect is that individuals frequently make choices that maintain the current state of the world (Masatlioglu & Ok, 2005; Samuelson & Zeckhauser, 1988).

In one study, researchers presented participants with products to purchase (e.g., music keyboards), and they varied the degree of information about the seller (Brown & Krishna, 2004). Some participants read generic information about the seller, whereas other participants read additional information about the seller having financial troubles and potentially going out of business. When participants were told this additional information about the seller, participants were more likely to switch away from the default. It seemed that participants interpreted the default as a recommendation from the seller, and when participants were concerned that the seller’s intentions did not align with customers’ interests, participants did not show a default effect. The same general tendency may occur for all default effects. People interpret the default as a recommendation from the experimenter or from an expert with whom interests are assumed to align, as people see these experts as trustworthy rather than self-interested, as in Brown and Krishna’s (2004) study.

### Framing Effects

Early theories on rational choice posited that human decision-making assumes the principle of invariance when making decisions involving making preferences among alternatives (Von Neumann & Morgenstern, 1944). According to this principle, individuals’ decisions are unaffected by the manner in which choices are articulated (termed description invariance) or by the procedure employed to gather preferences (known as procedural invariance). Seminal works by Daniel Kahneman, Amos Tversky, and their peers revealed that the assumptions of both description invariance and procedural invariance are frequently violated in human decision-making.

One type of framing effect investigates how procedures employed to gather preference affect decisions. That is, people’s decisions are influenced by the way a decision scenario is framed—whether that be by different wordings, settings, or situations (Brewer & Kramer, 1986; De Martino et al., 2006; Fagley & Miller, 1987; Gamliel & Kreiner, 2013; Levin & Gaeth, 1988; Piñon & Gambara, 2005; Puto, 1987; Rothman & Salovey, 1997). For example, presenting the same information on risks where the alternatives are described in positive terms (i.e., lives saved) or in negative terms (i.e., lives lost) can affect preferences (Tversky & Kahneman, 1981). A large body of literature shows that framing effects pervasively influence judgments and decisions (for review, see Levin et al., 1998).

Although there are many ways a decision scenario can be framed, one salient frame people often use with decision scenarios for arriving at their preferences among the alternatives can be to approach it as a *selection* or a *rejection* task. That is, individuals have the option to choose between two alternatives either by picking one (thereby indirectly discarding the other) or by dismissing one (thus indirectly keeping the other). Research, however, has uncovered that such decision-making scenarios can lead to diverging preferences for the same options (Shafir, 1993; Wedell, 1997).

The Choose versus Reject (also termed as Selection vs. Rejection) task frame is a type of framing effect that has helped us better understand people’s decisions when deciding between products and job applicants (Park et al., 2000; Sokolova & Krishna, 2016) and when choosing among products (Chernev, 2009; Nagpal & Krishnamurthy, 2008). There have been two independent theoretical mechanisms proposed in the literature to explain the framing effects. First, Shafir (1993) proposed a compatibility hypothesis where positive attributes are given greater emphasis during the choosing of an option, while negative attributes are more heavily considered during the rejection of an option. The compatibility hypothesis is an attentional mechanism arguing that the choose frame is accompanied by attention toward positive attributes as the task of choosing is to find good reasons to choose an option, whereas the reject frame is accompanied by attention toward negative attributes as the rejection task is to find flaws to reject an option.

Wedell (1997) proposed the accentuation hypothesis as an alternative theoretical mechanism. This theory posits a motivational mechanism suggesting that individuals exhibit more discernment in tasks involving choice, showing a tendency to favor the more appealing option more frequently and the less appealing option less frequently. Therefore, if one option is perceived to be more appealing than another, the likelihood of participants preferring the more attractive option increases in tasks that involve choosing as opposed to those that involve rejection. Unlike the theoretical assumption of the compatibility hypothesis taking the view that people pay more attention to positive or negative attributes based on the task frame, the accentuation hypothesis assumes that people make overall judgments of alternatives considering both positive and negative attributes associated with the alternatives. Wedell (1997) summarized it as “The accentuation hypothesis simply argues that greater commitment or need for justification in choice leads to greater weighting of attribute differences” (p. 874). Recent work comparing the compatibility versus accentuation hypothesis has found stronger support for the accentuation hypothesis (Chandrashekar et al., 2021).

Based on the accentuation hypothesis, we argue that decision-makers will exert greater effort in evaluating and discerning the relative advantages of alternatives in the choose decision frame than in the reject decision frame. To reiterate our theoretical prediction with an example: When participants are asked to indicate their preference between two job opportunities that have differing strengths and weaknesses, the job opportunity with a relatively stronger overall evaluation will be preferred more frequently in the *choose* decision frame than during the reject decision frame.

### Conceptual Similarity With Previous Work

Conceptually, the present study is close to Johnson et al. (2002). Johnson et al.’s (2002) scenario involved a simple Go/No Go choice between agreeing or disagreeing to receive marketing promotions via email. The current study has four important distinctions. First, our study scenarios involve substantive individual decisions, such as the choice between two jobs or the choice between two different medications. Second, the decision scenario involved a choice between two alternatives in contrast to Johnson et al.’s (2002) Go/No Go decision scenario. Third, the default was manipulated based on a broader definition of default in which a default alternative was endowed and also preselected as the answer option. Finally, Johnson et al. (2002) used the term “DO NOT send me…” as the negative framing, while we sought to test the efficacy of the terms accept and reject.

Given the distinct theoretical mechanisms argued as part of defaults and framing effects, there are two possibilities when considering the simultaneous influence of defaults and framing on choice. Johnson et al. (2002) reported additive influences of default and framing predictors. However, the replication of Johnson et al. (2002) by Chandrashekar et al. (2023) found a lack of support for the default predictor, whereas the framing effect was supported.

#### Hypotheses

The study was preregistered on the open science framework before data collection. Given the nature of the design, the authors of the study did not have concrete directional predictions. The following was one of the exploratory predictions related to the framing predictor:

Exploratory prediction: The proportion of people who stay with the given default option in a choice (“positive choice”) decision framing scenario will be different from the proportion of people who stay with the given default option in a reject (“negative choice”) decision framing scenario.

## Method

### Preregistration and Open Science

We preregistered the experiment on Open Science Framework. The preregistration, study materials, data, and R analysis code are available at: https://osf.io/uwm7r/.

### Supplementary Materials

In the supplementary materials section, we document the results based on the full sample of participants without exclusions, provide a summary of the a priori power analysis that formed a part of our preregistration, and include disclosures that summarize any deviations from the preregistered analysis.

### Ethical Approval

The study was approved by the ethical review committee at Hong Kong Metropolitan University. All the study procedures and methods noted in the manuscript are in accordance with the relevant guidelines and regulations. We invited participants older than 18 years. Informed consent was obtained from all participants of the study.

### Participants and Power Analysis

We expected to detect a critical *z* value of 1.96 (two proportions *z*-test comparing two between-subjects comparisons) of .16 with 80% power and an α = .05. We planned to recruit 175 participants per condition, for a total of 1,050 participants (across six conditions). The participants were recruited via the CloudResarch platform in October 2019. The data collection was conducted in October 2019. Altogether, 1,110 US participants completed the study.

As per our preregistration, participants were excluded from the main analyses if they met at least one of the following criteria: (1) They indicated a low proficiency in English (self-report < 5, on a 1–7 scale); (2) self-reported not being serious about filling in the survey (self-report < 4, on a 1–5 scale); (3) participants who reported that they have seen the questions before; (4) participants who correctly guessed hypotheses; (5) did not complete the study. To obtain the final sample, we first excluded 38 participants^2^ following our preregistered exclusion criteria, resulting in 1,072 participants (*M*_age_ = 39.95, *SD*_age_ = 12.00; 522 males, 545 females, five others).

### Procedure

The scenarios were adapted from previous research by Zlatev et al. (2017). The study design involved 2 Framing (Choose vs. Reject) × 3 Default (No-default vs. Default 1 vs. Default 2). The framing and default manipulations were between-subjects experimental conditions and scenarios were a within-subjects factor. The procedure involved two parts. In the first part, participants read about a job choice scenario, and in the second part, participants responded to a scenario involving a choice between two medications. Once a participant was allocated to one of the six between-subjects conditions, participants read the two decision scenarios from the same condition. The presentation order between the job choice scenario and medication scenario was not randomized, i.e., all participants first read the job scenario and then the medication scenario. We randomized the display of answer options across participants. After completing both parts of the survey, participants provided their demographic information, and they were debriefed at the end of the study.

Tables 1 and 2 carry the descriptions of the scenarios. Specifically, the job scenarios involved two jobs with their respective advantages and drawbacks, and participants had to make a selection between them. For example, selecting a job with more vacations in the choose condition meant preference toward this option, while in the reject condition was against this option.^3^ Similarly, the medication scenarios presented two medications along with their advantages and drawbacks, and participants had to indicate a preference between them. In part 1 of the experiment, participants in the No-default conditions were able to freely choose or reject one of the job alternatives based on their preference—i.e., without a preselected answer. On the other hand, participants assigned to any of the default conditions were informed that they were already employed in a job with a certain salary and holiday benefits. They were then given the opportunity to choose (or reject) between their current job and an alternative option.

**Table 1:** Job selection scenarios

	Choose	Reject
**No-default**	Imagine that you have recently graduated and are looking for a job. You currently have two options and have to decide between them based on the limited information shown below. Which one would you **CHOOSE?** o *A job that* • Pays **$600** per week • Offers you **20 paid vacation days** per year o *A job that* • Pays **$630** per week • Offers you **10 paid vacation days** per year	Imagine that you have recently graduated and are looking for a job. You currently have two options and have to decide between them based on the limited information shown below. Which one would you **REJECT?** o *A job that* • Pays **$600** per week • Offers you **20 paid vacation days** per year o *A job that* • Pays **$630** per week • Offers you **10 paid vacation days** per year
**Default condition 1: Vacation default**	Imagine that you have recently graduated and you have a job that pays $600 per week and offers you 20 paid vacation days per year. You can switch to another job in a different office of the same company, and the new job is the same as your current job, except for one distinct advantage and one distinct drawback: • The advantage is that the new job pays **$630 per week ($30 more per week).** • The drawback is that the new job offers you **only 10 vacation days per year.** Which one would you **CHOOSE?** o *Current job that pays $600 per week and offers 20 paid vacation days per year* o *New job that pays $630 per week and offers 10 paid vacation days per year*	Imagine that you have recently graduated and you have a job that pays $600 per week and offers you 20 paid vacation days per year. You can switch to another job in a different office of the same company, and the new job is the same as your current job, except for one distinct advantage and one distinct drawback: • The advantage is that the new job pays **$630 per week ($30 more per week).** • The drawback is that the new job offers you **only 10 vacation days per year.** Which one would you **REJECT?** o *Current job that pays $600 per week and offers 20 paid vacation days per year* o *New job that pays $630 per week and offers 10 paid vacation days per year*
**Default condition 2: Salary default**	Imagine that you have recently graduated and you have a job that pays $630 per week and offers you 10 paid vacation days per year. You can switch to another job in a different office of the same company, and the new job is the same as your current job, except for one distinct advantage and one distinct drawback: • The advantage is that the new job offers you 2**0 vacation days per year.** • The drawback is that the new job pays you only **$600 per week ($30 less per week).** Which one would you **CHOOSE?** o *Current job that pays $630 per week and offers 10 paid vacation days per year* o *New job that pays $600 per week and offers 20 paid vacation days per year*	Imagine that you have recently graduated and you have a job that pays $630 per week and offers you 10 paid vacation days per year. You can switch to another job in a different office of the same company, and the new job is the same as your current job, except for one distinct advantage and one distinct drawback: • The advantage is that the new job offers you 2**0 vacation days per year.** • The drawback is that the new job pays you only6 **$600 per week ($30 less per week).** Which one would you **REJECT?** o *Current job that pays $630 per week and offers 10 paid vacation days per year* o *New job that pays $600 per week and offers 20 paid vacation days per year*

**Table 2:** Medication scenarios

	Choice	Reject
**No-default**	Imagine that you need to take a medication, and you have two options described below. Which medication would you **CHOOSE?** o ***Medication A*** • The advantage of Medication A is that it costs $20 per month. • The drawback of Medication A is that it needs to be taken 6 times a week. o ***Medication B*** • The advantage of Medication B is that it needs to be taken only once per week. • The drawback of Medication B is that it costs $50 per month.	Imagine that you need to take a medication, and you have two options described below. Which medication would you **REJECT?** o ***Medication A*** • The advantage of Medication A is that it costs $20 per month. • The drawback of Medication A is that it needs to be taken 6 times a week. o ***Medication B*** • The advantage of Medication B is that it needs to be taken only once per week. • The drawback of Medication B is that it costs $50 per month.
**Default condition 1: Frequency default**	Imagine you are a sick patient and you are currently taking the generally prescribed medication, which costs $50 per month and needs to be taken once a week. You can switch to another medication. This new medication is nearly the same as the current medication, except for one distinct advantage and one distinct drawback: • The advantage is that the new medication costs **$20 per month.** • The drawback is that the new medication needs to be taken **6 times a week.** Which medication would you **CHOOSE?** o *Medication costing* ***$50 per month*** *and needs to be taken* ***once a week***. o *Medication costing* ***$20 per month*** *and needs to be taken* ***6 times a week***.	Imagine you are a sick patient and you are currently taking the generally prescribed medication, which costs $50 per month and needs to be taken once a week. You can switch to another medication. This new medication is nearly the same as the current medication, except for one distinct advantage and one distinct drawback: • The advantage is that the new medication costs **$20 per month.** • The drawback is that the new medication needs to be taken **6 times a week.** Which medication would you **REJECT?** o *Medication costing* ***$50 per month*** *and needs to be taken* ***once a week***. o *Medication costing* ***$20 per month*** *and needs to be taken* ***6 times a week***.
**Default condition 2: Cost default**	Imagine you are a sick patient and you are currently taking the generally prescribed medication, which costs $20 per month and needs to be taken 6 times a week. You can switch to another medication. This new medication is nearly the same as the current medication, except for one distinct advantage and one distinct drawback: • The advantage is that the new medication needs to be taken **once a week**. • The drawback is that the new medication costs **$50 per month.** Which medication would you **CHOOSE?** o *Medication costing* ***$20 per month*** *and needs to be taken* ***6 times a week***. o *Medication costing* ***$50 per month*** *and needs to be taken* ***once a week.***	Imagine you are a sick patient and you are currently taking the generally prescribed medication, which costs $20 per month and needs to be taken 6 times a week. You can switch to another medication. This new medication is nearly the same as the current medication, except for one distinct advantage and one distinct drawback: • The advantage is that the new medication needs to be taken **once per week.** • The drawback is that the new medication is that it costs **$50 per month.** Which medication would you **REJECT?** o *Medication costing* ***$20 per month*** *and needs to be taken* ***6 times a week***. o *Medication costing* ***$50 per month*** *and needs to be taken* ***once a week.***

### Analysis

Data were analyzed using R (R Core Team, 2023). We conducted two separate analyses matching the responses across two scenarios. Data were fit to logistic regression models using the *glm* function (with “binomial(‘logit’)” as the family).

When analyzing the responses to the job scenario, a 2 × 3 binomial logistic regression was performed. The predictors included framing (Choose vs. Reject), default conditions (No-Default, Default 1, Default 2), and the interaction terms between framing and defaults (see Equation 1). These predictors were used to predict the respondent’s final preference, where 1 represented a job that paid $600/week and offered 20 days of paid vacation a year, and 0 represented a job that paid $630/week and offered 10 days of paid vacation a year.

Binary preference=α+β1(Framing)+β2(Default:Opt−out vs. Opt−in)+β3(Default:Opt−out vs. no default)+β4(Framing×Opt−out vs. Opt−in)+β5(Framing×Opt−out vs. no default)+ε.

Similarly, the analysis based on the responses to the medication scenario involved the same set of predictors to predict the respondent’s final preference. In this case, one represented a medication costing $20 per month and needing to be taken 6 times a week, while 0 represented a medication costing $50 per month and needing to be taken once a week.

## Results

### Descriptives

**Figure 1:**
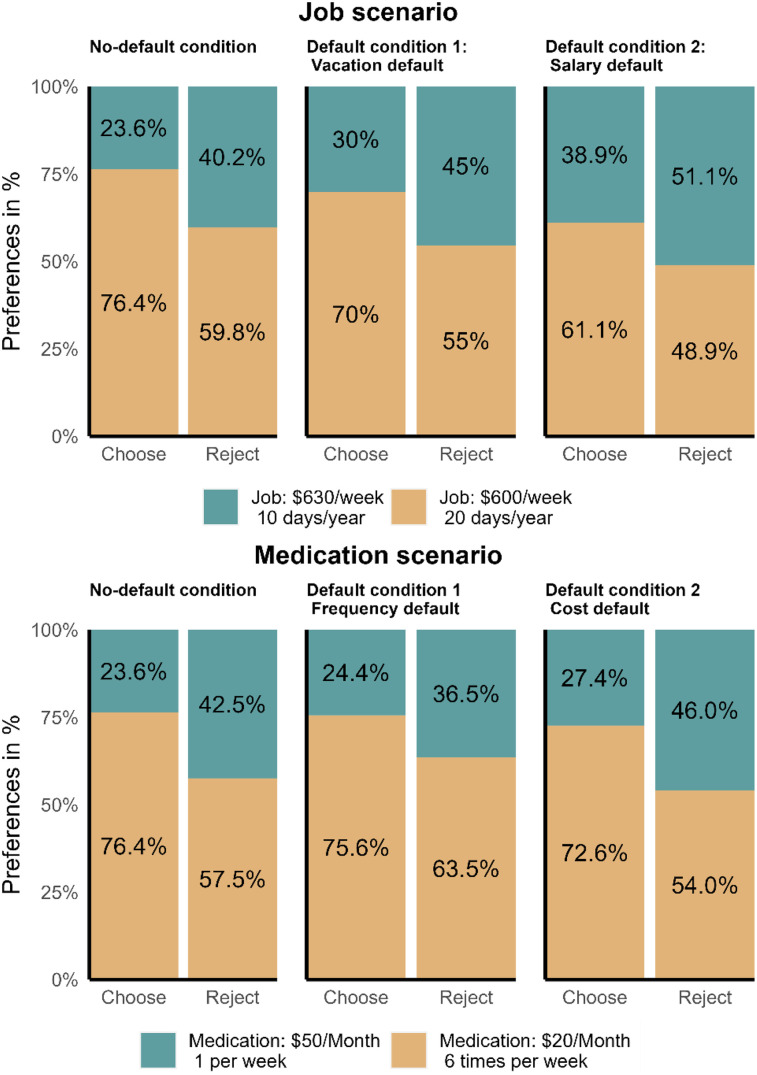
Responses to job and medication scenarios across the framing and default conditions.

We found that the majority of participants in no-default conditions preferred the job alternative with more paid vacation days in the job scenario and cheaper medication alternatives in the medication scenario (see Figure 1).

### Main Effects

#### Job Scenario

Concerning the job scenarios, the results are based on a logistic model (estimated using the Maximum Likelihood method) to predict job preference with Framing and Default conditions. We found support for framing as a predictor (odds ratio (*OR*) = 0.46, 95% CI [0.29, 0.72], *p* < .001). Additionally, Default condition 2 was also a significant predictor (*OR* = 0.49 [0.31, 0.76], *p* = .002).

The results suggest that participants in the choose framing condition chose the job with a larger number of paid vacation days (69.1%; averaging across three default conditions) at a higher rate than the participants in the reject framing condition (54.4%; also see Figure 1 and Table 3). We looked at the contrasts between choosing and rejecting within each condition separately and found similar patterns within each condition [No-default: χ^2^(1) = 11.45, *p* < .001, *V*_Cramer_ = 0.17, 95% CI [0.07, 1.00], *n*_obs_ = 361; Default 1 (Vacation default): χ^2^(1) = 8.72, *p* < .001, *V*_Cramer_ = 0.15, 95% CI [0.04, 1.00], *n*_obs_ = 353; Default condition 2 (Salary default): χ^2^(1) = 5.41, *p* = .020, *V*_Cramer_ = 0.11, 95% CI [0.00, 1.00], *n*_obs_ = 358].

**Table 3:** Job scenario: results based on logistic regression analysis with interaction terms

Predictors	Job preference				
	Odds ratios	*SE*	CI	Statistic	*p*
(Intercept)	3.23	0.56	2.32–4.60	6.72	**<.001**
Framing (Reject vs. Choose)	0.46	0.11	0.29–0.72	−3.35	**.001**
Default condition 1 (DC1)	0.72	0.17	0.45–1.14	−1.40	.162
Default condition 2 (DC2)	0.49	0.11	0.31–0.76	−3.11	**.002**
Framing × DC1	1.13	0.36	0.60–2.13	0.38	.704
Framing × DC2	1.32	0.42	0.71–2.46	0.89	.375
Observations	1,072
*R*^2^ Tjur	0.035
*Note*. Default 1 condition had the job with higher vacation days as the preselected option and Default 2 condition had higher paid job as the preselected option.					

#### Medication Scenario

Concerning the Medication scenarios, our results indicated support for framing effect (*OR* = 0.42 [0.27, 0.66], *p* < .001). The results suggest that participants in the choose framing condition chose the cheaper medication at a higher rate (74.9%; averaging across three default conditions) than the participants in the reject framing condition (58.4%; also see Figure 1 and Table 4). We looked at the contrasts between choosing and rejecting within each condition separately and found similar patterns within each condition [No-default: χ^2^(1) = 14.48, *p* < .001, *V*_Cramer_ = 0.19, 95% CI [0.10, 1.00], *n*_obs_ = 361; Default 1 (Cost default): χ^2^(1) = 6.16, *p* = .008, *V*_Cramer_ = 0.12, 95% CI [0.00, 1.00], *n*_obs_ = 358; Default 2 (Frequency default): χ^2^(1) = 13.17, *p* < .001, *V*_Cramer_ = 0.19, 95% CI [0.09, 1.00], *n*_obs_ = 353].

**Table 4:** Medication scenario: results based on logistic regression analysis

Predictors	Medication preference				
	Odds ratios	*SE*	CI	Statistic	*p*
(Intercept)	3.23	0.56	2.32–4.60	6.72	**<.001**
Framing (Reject vs. Choose)	0.42	0.10	0.27–0.66	−3.76	**<.001**
Default condition 1 (DC1)	0.96	0.24	0.59–1.55	−0.18	.855
Default condition 2 (DC2)	0.82	0.20	0.51–1.32	−0.82	.414
Framing × DC1	1.34	0.44	0.71–2.55	0.90	.370
Framing × DC2	1.06	0.34	0.56–1.99	0.17	.865
Observations	1,072
*R*^2^ Tjur	0.035
*Note*. Medication scenario: Default 1 condition had expensive medication as the preselected option and Default 2 condition had cheaper medication as the preselected option.					

## Discussion

This study indicates differential effects for both default and framing on decision-making. The results suggest that framing manipulations have a meaningful influence on decision-making processes, altering the way individuals perceive and respond to change in their lives.

Framing manipulation had a strong influence on the choice, as participants in the positive framing (i.e., choose) decided to go with a dominant choice to the same extent as in the condition without prior, while participants in the negative framing (i.e., reject) decided to stick to a dominant choice to a lesser extent. Default also influenced the choice, but it seems that the framing effect is stronger than the default effect. Indeed, in the no prior condition, overall participants preferred more vacation (68%) over salary (32%) and a less expensive but more cognitively demanding medication (67%) over more expensive but less cognitively demanding medication (33%).

Comparing the size of the effect, framing had a stronger effect than default on participants' choices. For example, a job with more vacation days and cheap medication were the dominant choices across different default conditions, but they consistently varied across the framing of the choices. The results seem to suggest that when given a choice frame, participants make an active preference toward a predominant choice. However, the preferences across the two alternatives are not clearly demarcated within reject conditions.

### Implication for the Theory

Johnson et al. (2002) attempted to compare the default effect and the frame effect and reported additive effects. However, a close replication of their work indicates that the framing effects were a stronger predictor than the default effects (Chandrashekar et al., 2023). The two decision scenarios included in the current study are very different from organ donation. For organ donation, the choice is for or against and includes a decision in the moral domain. The no-default choice is “Please choose your preferred organ donor status – yes or no”. In the case of our experiments, both options offer advantages and disadvantages. If participants prefer more vacations to better pay or prefer a medication that is easier to take but more expensive, this reflects an individual choice, closer to those made by individuals more frequently than the choice of organ donation. These are also individual choices and not entirely prosocial decisions and therefore represent a different decision context (Krishnamurthy et al., 2001). For these reasons, our experiment goes beyond previous work and sheds new light on the ways in which the presentation of choices can modify people's decisions.

The current findings also underline the robustness of framing effects, a finding already acknowledged in several studies on the framing effect (Chandrashekar et al., 2021; Chen & Proctor, 2017; Wedell, 1997). The current findings suggest support for the accentuation hypothesis, which argues that the choose frame led to higher motivational engagement. The pattern of the results we observe indicates that there is a greater frequency of preference toward a normative choice or dominant alternative. In our case, it was the job with more vacation days in the job scenario and the cheaper medication in the medication scenario. However, we did not fully test the tenets of the accentuation theory by varying the relative attractiveness of the alternatives within each scenario. Thus, our findings do not fully rule out the compatibility account.

We found that the results of the present study are aligned with the replication from Chandrashekar et al. (2023), indicating support for the framing predictors but no support for default predictors. Why do we find support for the default predictor in the job scenario but not the medication scenario? The two scenarios are qualitatively different. We speculate that participants may have paid more attention during the medication scenario as it is related to their health and well-being.

### Limitations

We also note limitations that suggest promising directions for future research. The primary limitation of our study is that our findings are based on a limited set of hypothetical dilemmas. As such, the applicability of our results to real decisions remains uncertain. Future studies should expand on this work and explicitly test whether our results hold up in actual decisions people make.

The decision scenarios involved in our study are limited to simple trade-offs. For example, in the job scenario, alternatives involved the comparisons of 10 additional days of holidays versus 30$ extra per week. The scenarios were adapted from previous research by Zlatev et al. (2017). For example, 35$ extra per week instead of 30$ extra per week might affect the findings and the conclusions we draw from those findings. However, we do not view this as a major issue as these relative trade-offs determine a normative choice. Therefore, the current findings shed light on the relative influence of framing and defaults given a relatively dominant alternative. Future work can rigorously examine the validity of our findings while varying the degree of dominance among alternatives. Such investigations can also test the specific theoretical mechanisms associated with the framing effects (accentuation vs. compatibility hypothesis). The findings are based on a convenient sample that involved participants from the Amazon Mechanical Turk platform, with documented limitations (Eyal et al., 2022). Future research should consider a more diverse and representative sample to enhance the generalizability of the findings.

All the participants in the study first responded to the job scenario followed by the medication scenario. It is possible that the nature of the description and responses during the first scenario may have influenced how participants answered the second scenario. However, we do not view this to be a serious concern. First, the scenarios were distinct from each other, coming from different domains. Second, we conducted a robustness check to ascertain if the participants who were exposed to default nudge in the first scenario and stayed with the default answer (or not) affected the pattern of responses in the second scenario. We did not find any support for a possibility that the pattern of responses in the first scenario relates to participants’ choice in the subsequent scenario (see Table S6 of the supplementary section for details of analysis).

The response to the scenarios might have been driven by participants’ personal preferences. The fact that the two scenarios (job and medication) lead to the same pattern helps ensure that personal preference has not influenced too much the choice made in these scenarios. Thus, it does not take away the main insight from the current finding that framing of the decision context can modulate the preferences. For example, although a majority of the participants across the experimental conditions preferred a job with more vacation days, and a cheaper medication, the degree of skew toward the majority varied based on the framing of the decision. Future studies can take into account personal preference by asking participants about their habits and preferences before conducting the survey.

Finally, as our methodology and decision scenarios were new and aimed toward an attempt to tackle the literature on mixtures of nudges, our preregistered hypothesis was nondirectional. Future studies can increase the preciseness of the scope by creating direct and conceptual replications testing the potential effects of using several types of nudges.

### Implications for the Practice

Scaling up nudges is the biggest challenge in practice. Dr. Elizabeth Linos noted at the 2023 Nudges in Health Care Symposium “When your goal is to change policy and implement nudges, you need to include the policy changers in the room at the design of nudge experiment/pilot” (Linos, 2023). We cannot rely on a single bias to influence desirable behavior. Nor can we assume that nudges will have an additive effect if we use several of them. Finally, we cannot assume that a cognitive bias will act in the same way in all contexts or on different choices. As our study has shown, the domain of influence, whether work or health, modifies the decision-making context and thus the influence of potential nudges. Our study points to three practical limitations and implications, as well as ways of improving experimental design.

Default nudge does not seem to be the most relevant nudge in our experiment, as it is in that of Chandrashekar et al. (2023). Supporting this view, in a recent meta-analysis of nudges (Maier et al., 2022; Mertens et al., 2022; Szaszi et al., 2022), 223 of the 447 nudges tested were default nudges and did not show any meta-analytical effect different from the null. In a second meta-analysis on the transparency of nudges, 105 of 114 effects concerned default nudges (Bruns et al., 2024). There is therefore an over-representation of this type of nudge, which is unreasonable considering its relative and contextual effectiveness.

An important aspect of the nudge’s philosophy is its ability to allow free, voluntary choice (De Ridder et al., 2022). In this sense, it must be legitimate and accepted by the population. A nudge by default only partly fulfils the contract: The experimenter or policymaker chooses the individual and asks them if they ultimately want to go back on the choice they did not make. On the other hand, a change of frame does not mean that the policymaker or experimenter has to speak for the individual, leaving them more room to decide. A framing nudge is therefore less paternalistic than a default nudge and could be more readily accepted, as well as being more effective in some cases. It remains necessary to test individual preferences for particular types of nudges in particular contexts, an area of research often neglected so far.

Finally, it is important to note that frame nudge also poses difficulties. First, it is very rare to answer questions that involve rejecting a proposition, which may have surprised participants, and the result may have more to do with a surprise effect than a frame effect. It can also be assumed that this formulation is more difficult to understand than a positive one, which may prejudice people with language or cognitive impairments. This debate is extremely current, given recent advances in the foreign language effect (Białek, 2023; Borkowska et al., 2023; McFarlane et al., 2020), a cognitive bias used as a nudge consisting of thinking in another language to make more rational choices. Studies are underway to determine the effectiveness of this type of nudge. It remains to be seen whether the framing effect, however effective it may be under controlled conditions, can be applied in the field setting.

### Conclusion

In an experiment involving a change of job and medication intake, we discovered that the frame effect and the default effect apply differently depending on the context. In both experiments, the default effect was weak to non-existent, showing that in some cases, the frame effect is stronger. This result is in line with recent meta-analyses on the low effectiveness of this type of nudge. Future work on default effects should be aware that people’s decision frame can reduce its effectiveness. These findings contribute to a growing body of literature emphasizing the malleability of cognitive biases and provide valuable insights for choice-architecture interventions. The current work contributes to understanding the generalizability of the findings and thus informs scientific scholars, Nudge Units, and practitioners to help people improve the choices they make about their lives.

## Electronic Supplementary Materials

The electronic supplementary materials are available with the online version of the article at https://doi.org/10.1027/1618-3169/a000617

**ESM 1.** Disclosures and additional results.

## Footnotes

^1^
Nudge Units are specialized teams within governments that leverage behavioral science insights to design subtle interventions or ”nudges,” aiming to positively influence individuals' decisions and behaviors while preserving freedom of choice.

^2^
We sequentially excluded 12, 15, and 11 participants based on exclusion Criteria 1, 2, and 3, respectively. There were no exclusions based on Criteria 4 and 5.

^3^
As part of the data analysis, the responses were recorded across conditions and scenarios, such that when a participant’s response suggested preference for a job offering higher vacation or a cheap medication the response was coded as “1,” otherwise, the response was coded as “0.” Please refer to the R analysis scripts on the OSF page for the details.

## Notes

**Publication Ethics:** The study was approved by the ethical review committee at Hong Kong Metropolitan University.

**Authorship:** Subramanya P. Chandrashekar, Conceptualization, Data curation, Funding acquisition, Methodology, Project administration, Software, Super vision. Subramanya P. Chandrashekar and Adrien Fillon, Formal analysis, Investigation, Resources, Validation, Visualization, Writing – original draft, Writing – review and editing.

**Open Science:** The manuscript includes a DOI generated by a public data repository pointing to the raw data underlying the findings reported in the article. The pre-registration, study materials, data, and R analysis code are available at: https://osf.io/uwm7r/ (Chandrasekar & Fillon, 2024).

The manuscript contains at least one experiment with a completely executed preregistration or explicit notes on any deviation from the preregistration.
