# Supplementary material for: Framing the Default: Influence of Choosing Versus Rejecting Frame on Default Effects
Source: Exp Psychol. 2024 Oct 9;71(3):164–75. doi: 10.1027/1618-3169/a000617 (PMC11612645; doi:10.1027/1618-3169/a000617)
Supplement: Supplementary file 1 [file zea_71_3_164_esm1.pdf]

Supplementary Information for:

Framing the default: Influence of Choosing vs Rejecting Frame on Default Effects

Table of contents

Disclosures 3

Disclosures Deviations from pre-registration 4

Additional results 5

Appendix A 10

Disclosures

Pre-registrations
Pre-registrations were conducted prior to data collection.

Data collection
Data collection was completed before conducting data analysis.

Conditions reporting
All collected conditions are reported.

Data exclusions
For our data analysis, we used the full sample size in order to obtain a result with higher generalizability. The results did meaningfully change with exclusion based on the pre-registered exclusion criteria.

Variable reporting
All variables collected as part of the study are reported and included in the provided data.

Power analysis.
We aimed to have 175 participants per experimental condition. The study design has 6 between-subject conditions (total N ~ 1050 participants). This sample size gives us enough power to detect critical z value of 1.96 (two proportions z-test comparing two between-subjects comparisons) of .16 with .80 power. The power analysis was conducted with G*Power 3.1.9.2 and the details of the power analysis (see Appendix A).

Disclosures Deviations from pre-registration

Table E1. Deviations from pre-registration

Components of pre-registration	Were there deviations?	If yes describe the details of the deviation(s)	Rationale for deviation	Additional notes
**Procedures**	No	N/A	N/A	N/A
**Power analysis**	No	N/A	N/A	N/A
**Exclusion rules**	No	N/A	N/A	N/A
**Evaluation criteria**	No	N/A	N/A	N/A
**Predictions**	No	N/A	N/A	N/A
**Analysis**	Yes	Pre-registration assumed a series of chi-squared tests for each of the two scenarios.<br><br>We conducted two binomial logistic regression analyses. We conducted 2x3 binomial logistic regression. The predictors included framing (Select vs. Reject), default conditions (No-Default, Default 1, Default 2), and the interaction terms between framing and defaults as the predictors of binary choice.	The binomial logistic regression analyses allow us to account for both framing and default influences on the choice.	

Additional results after exclusions

Table E2

Job Scenario: Results based on logistic regression analysis without interaction terms

Predictors	Job preference				
	Odds Ratios	*S.E.*	*CI*	*Z*	*p*
(Intercept)	2.99	0.40	2.30 – 3.90	8.13	**<0.001**
Framing [Reject vs. Choose]	0.53	0.07	0.41 – 0.68	-4.96	**<0.001**
Default condition 1 (DC1)	0.77	0.12	0.56 – 1.05	-1.66	0.096
Default condition 2 (DC2)	0.56	0.09	0.41 – 0.77	-3.64	**<0.001**
Observations	1072				
R^2	0.035				

Note. N = 1072. Default 1 condition had the job with higher vacation days as the preselected option and Default 2 condition had the higher paid job as the preselected option.

Table E3

Job Scenario: Results based on logistic regression analysis with interaction terms

	Job preference				
Predictors	*Odds Ratios*	*std. Error*	*CI*	*Statistic*	*p*
(Intercept)	3.23	0.56	2.32 – 4.60	6.72	**<0.001**
Framing [Reject vs. Choose]	0.46	0.11	0.29 – 0.72	-3.35	**0.001**
Default condition 1 (DC1)	0.72	0.17	0.45 – 1.14	-1.40	0.162
Default condition 2 (DC2)	0.49	0.11	0.31 – 0.76	-3.11	**0.002**
Framing × DC1	1.13	0.36	0.60 – 2.13	0.38	0.704
Framing × DC2	1.32	0.42	0.71 – 2.46	0.89	0.375
Observations	1072				
R^2 Tjur	0.035				

Note. N = 1072. Default 1 condition had the job with higher vacation days as the preselected option and Default 2 condition had the higher paid job as the preselected option.

Table E4

Medication Scenario: Results based on logistic regression analysis without interaction terms

	Medication preference				
Predictors	*Odds Ratios*	*std. Error*	*CI*	*Statistic*	*p*
(Intercept)	3.03	0.41	2.33 – 3.97	8.16	**<0.001**
Framing [Reject vs. Choose]	0.47	0.06	0.36 – 0.61	-5.69	**<0.001**
Default condition 1 (DC1)	1.13	0.18	0.82 – 1.55	0.74	.458
Default condition 2 (DC2)	0.85	0.14	0.62 – 1.16	-1.03	.301
Observations			1072		
R^2 Tjur			0.034		

Note. N = 1072. Medication scenario: Default 1 condition had expensive medication as the preselected option and Default 2 condition had the cheaper medication as the preselected option.

Table E5

Medication Scenario: Results based on logistic regression analysis with interaction terms

	Medication preference				
Predictors	*Odds Ratios*	*std. Error*	*CI*	*Statistic*	*p*
(Intercept)	3.23	0.56	2.32 – 4.60	6.72	**<0.001**
Framing [Reject vs. Choose]	0.42	0.10	0.27 – 0.66	-3.76	**<0.001**
Default condition 1 (DC1)	0.96	0.24	0.59 – 1.55	-0.18	.855
Default condition 2 (DC2)	0.82	0.20	0.51 – 1.32	-0.82	.414
Framing × DC1	1.34	0.44	0.71 – 2.55	0.90	.370
Framing × DC2	1.06	0.34	0.56 – 1.99	0.17	.865
Observations			1072		
R^2 Tjur			0.035		

Note. N = 1072. Medication scenario: Default 1 condition had expensive medication as the preselected option and Default 2 condition had the cheaper medication as the preselected option.

Table E6

Medication Scenario: Robustness check

	Medication preference					Medication preference				
Predictors	*Odds Ratios*	*std. Error*	*CI*	*Statistic*	*p*	*Odds Ratios*	*std. Error*	*CI*	*Statistic*	*p*
(Intercept)	3.09	0.54	2.22 – 4.39	6.51	**<0.001**	3.08	0.57	2.16 – 4.47	6.08	**<0.001**
Framing	0.56	0.13	0.35 – 0.89	-2.47	**.014**	0.56	0.13	0.35 – 0.89	-2.47	**.014**
Default	0.86	0.21	0.53 – 1.38	-0.63	.527	0.86	0.21	0.53 – 1.39	-0.63	.526
Framing × Default	0.79	0.26	0.42 – 1.49	-0.74	.462	0.79	0.26	0.41 – 1.51	-0.71	.476
Default response in the preceding scenario (Yes vs. No)						1.01	0.17	0.73 – 1.40	0.07	.944
Observations	711					711				
R^2 Tjur	0.032					0.032				

Note. The robustness analysis noted in the table only includes responses from the default conditions.

Additional results based on FULL Sample

Table E7

Descriptive table of the participants' preferences.

	**Default condition**	**Framing condition**	***n***	**Preference for a job offering higher vacation days (in %)**
**Job scenario**	No-default	Choose	186	76%
		Reject	183	60%
	Default condition 1: Vacation default	Choose	183	69%
		Reject	184	55%
	Default condition 2: Salary default	Choose	186	61%
		Reject	188	49%

	**Default condition**	**Framing condition**	***n***	**Preference for the cheaper medicine alternative (in %)**
**Medication Scenario**	No-default	Choose	186	77%
		Reject	183	57%
	Default condition 1: Cost default	Choose	186	75%
		Reject	188	63%
	Default condition 2: Frequency default	Choose	183	73%
		Reject	184	54%

Note. $N = 1110$. Job scenario: Default 1 condition had the job with higher vacation days as the preselected option and Default 2 condition had the higher salaried job as the preselected option; Medication scenario: Default 1 condition had expensive medication as the preselected option and Default 2 condition had the cheaper medication as the preselected option.

Table E8

Job Scenario: Results based on logistic regression analysis without interaction terms

Predictors	Job preference				
	Odds Ratios	*S.E.*	*CI*	*Z*	*p*

(Intercept)	2.97	0.39	2.30 – 3.87	8.20	**<0.001**
Framing [Reject vs. Choose]	0.54	0.07	0.42 – 0.69	-4.87	**<0.001**
Default condition 1 (DC1)	0.76	0.12	0.56 – 1.03	-1.75	.080
Default condition 2 (DC2)	0.56	0.09	0.41 – 0.75	-3.77	**.001**
Observations			1110		
R^2			0.034		

Note. N = 1110. Default 1 condition had the job with higher vacation days as the preselected option and Default 2 condition had higher paid job days as the preselected option.

Table E9

Medication Scenario: Results based on logistic regression analysis without interaction terms

Predictors	Job preference				
	Odds Ratios	*S.E.*	*CI*	*Z*	*p*
(Intercept)	3.08	0.41	2.37 – 4.02	8.35	**<0.001**
Framing [Reject vs. Choose]	0.46	0.06	0.36 – 0.60	-5.88	**<0.001**
Default condition 1 (DC1)	1.09	0.17	0.80 – 1.50	0.56	.575
Default condition 2 (DC2)	0.85	0.13	0.62 – 1.15	-1.05	.293
Observations			1110		
R^2			0.034		

Note. N = 1110. Medication scenario: Default 1 condition had expensive medication as the preselected option and Default 2 condition had the cheaper medication as the preselected option.

Appendix A

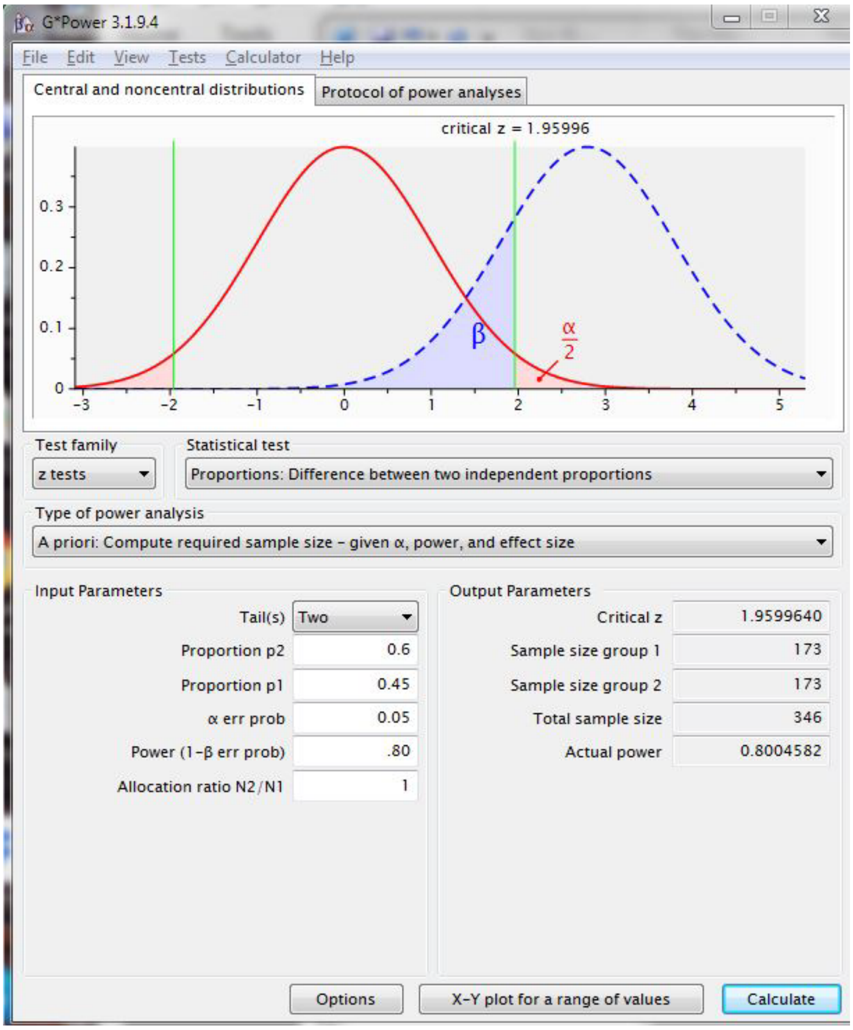
G*Power 3.1.9.4
File Edit View Tests Calculator Help
Central and noncentral distributions
Protocol of power analyses
critical z = 1.95996
0.3
0.2
0.1
0
−3 −2 −1 0 1 2 3 4 5
β
α/2
Test family
z tests
Statistical test
Proportions: Difference between two independent proportions
Type of power analysis
A priori: Compute required sample size – given α, power, and effect size
Input Parameters
Tail(s) Two
Proportion p2 0.6
Proportion p1 0.45
α err prob 0.05
Power (1−β err prob) .80
Allocation ratio N2/N1 1
Output Parameters
Critical z 1.9599640
Sample size group 1 173
Sample size group 2 173
Total sample size 346
Actual power 0.8004582
Options
X-Y plot for a range of values
Calculate
